# Reflecting on Fleming’s caveat: the impact of stakeholder decision-making on antimicrobial resistance evolution

**DOI:** 10.1099/mic.0.001534

**Published:** 2025-02-26

**Authors:** Tom Ashfield, Mineli Cooray, Isabel Jimenez-Acha, Zeshan Riaz, Danna R. Gifford, Mato Lagator

**Affiliations:** 1 The Signpost, Winchester, Hampshire, UK; 2 School of Life Sciences, University of Warwick, Warwickshire, UK; 3 Division of Evolution, Infection and Genomics, School of Biological Sciences, Faculty of Biology, Medicine and Health, The University of Manchester, Manchester, Greater Manchester, UK; 4 Medical Affairs, Specialty Care Division, Pfizer, Tadworth, Surrey, UK

**Keywords:** antimicrobial resistance, global response to antimicrobial resistance (AMR), policy, stewardship

## Abstract

Antimicrobial resistance poses one of the greatest and most imminent threats to global health, environment and food security, for which an urgent response is mandated. Evolutionary approaches to tackling the crisis tend to focus on proximate issues including the mechanisms and pathways to resistance, with associated calls to action for infection control and antimicrobial stewardship. This is of clear benefit but overlooks the fundamental influence of policy and stakeholder decision-making on resistance evolution. In 1945, Fleming issued a stark warning on the irresponsible use of penicillin and its potential to cause death due to penicillin-resistant infections. Attention to resistance evolution theory and heeding Fleming’s advice could have allowed for a vastly different reality. Embedding evolutionary theory within policy, industry and regulatory bodies is not only essential but is now a race against time. Hence, critical appraisal of historical behaviour and attitudes at a global scale can inform a paradigm of anticipatory and adaptive policy. To undertake this exercise, we focused on the largest group of antibiotics with the greatest clinical and economic footprint, the beta-lactams. We examined historical case studies that affected how beta-lactams were developed, produced, approved and utilized, in order to relate stakeholder decision-making to resistance evolution. We derive lessons from these observations and propose sustainable approaches to curb resistance evolution. We set a position that actively incorporates an evolutionary theory of antimicrobial resistance into decision-making within antimicrobial development, production and stewardship.

## Introduction

Antimicrobial resistance (AMR) poses one of the greatest threats to healthcare systems globally [1] and has been dubbed a ‘silent pandemic’ [2]. It is set to be one of the highest causes of global mortality by 2050 [3] with 4.95 million bacterial AMR-associated deaths occurring in 2019 alone [4]. Antimicrobials enable modern healthcare provision and have an immeasurable impact on reducing the global health burden of infection, yet current economic and societal valuation methods do not fully capture this. In a post-antibiotic era, procedures such as chemotherapy and surgery would carry unacceptably high risks due to the mortality associated with infection [5,6]. Those who are immunocompromised stand to be the worst affected [7]. Hence, AMR requires an urgent call to action – one that is currently lacking full global cohesion [8].

The first documented instance of AMR predated the discovery of penicillin through observations with Salvarsan in 1924 Prussia – Alexander Fleming’s discovery would not occur until 1928 [9]. Later, in 1945, Fleming stated a clear caveat following the global commercialized production of penicillin, framing today’s stark need for an orchestrated, global response to AMR:

'The greatest possibility of evil in self-medication [with penicillin] is the use of too-small doses, so that, instead of clearing up the infection, the microbes are educated to resist penicillin and a host of penicillin-fast organisms is bred out which can be passed on to other individuals and perhaps from there to others until they reach someone who gets a septicaemia or a pneumonia which penicillin cannot save. In such a case the thoughtless person playing with penicillin treatment is morally responsible for the death of the man who finally succumbs to infection with the penicillin-resistant organism. I hope this evil can be averted.'

Alexander Fleming, *New York Times*, 26 June 26, 1945

**Fig. 1.**
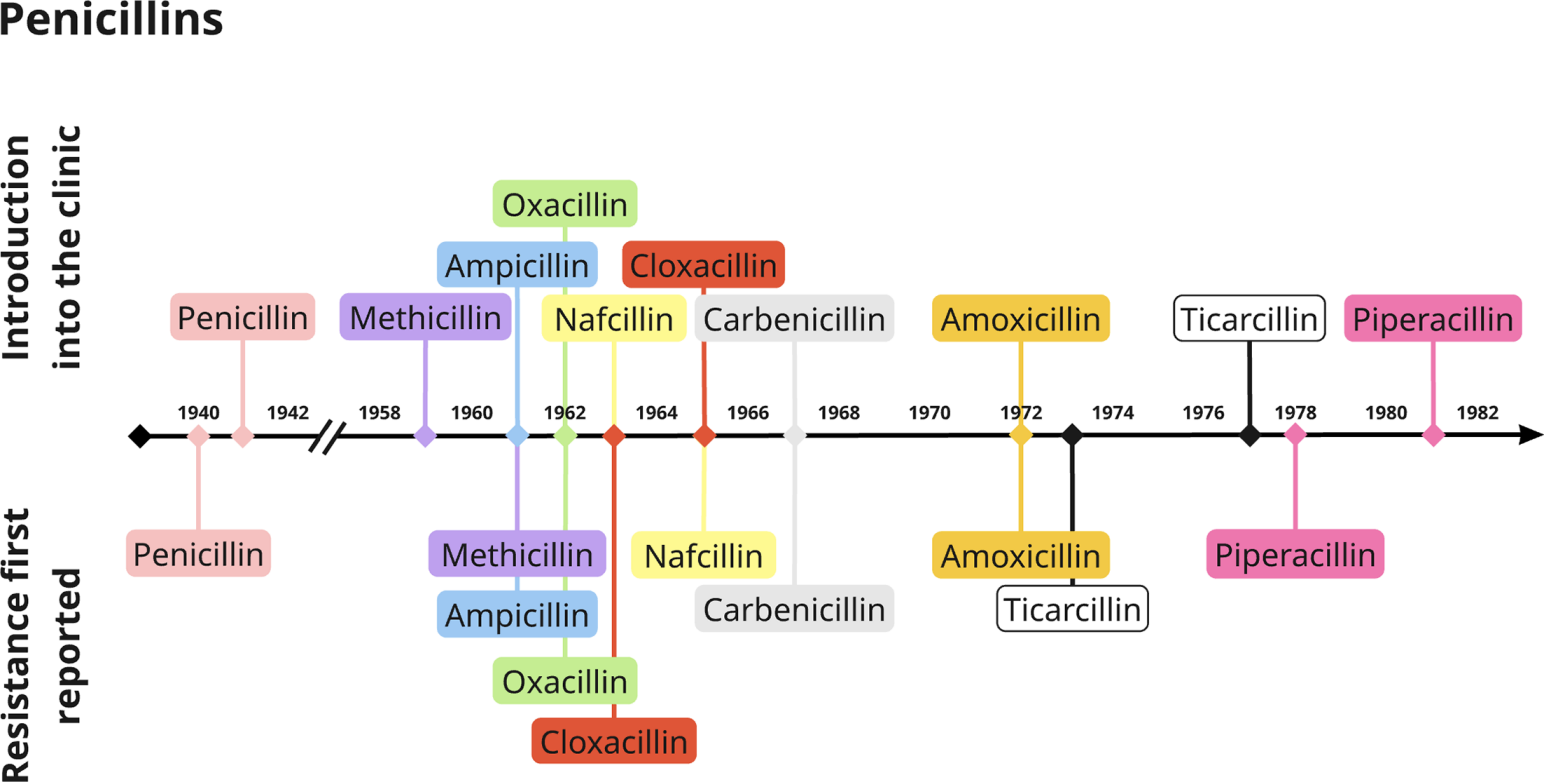
Timeline of penicillin introduction into clinical use and the first reports of resistance. For each antibiotic, we searched PubMed and Google Scholar for the first report of its clinical use or approval (top section of the plot) and the first report of resistance either in a clinic or laboratory setting (bottom section). Resistance was defined when a strain evolved resistance to an antibiotic to which it was previously sensitive. Colours indicate the antibiotic.

**Fig. 2.**
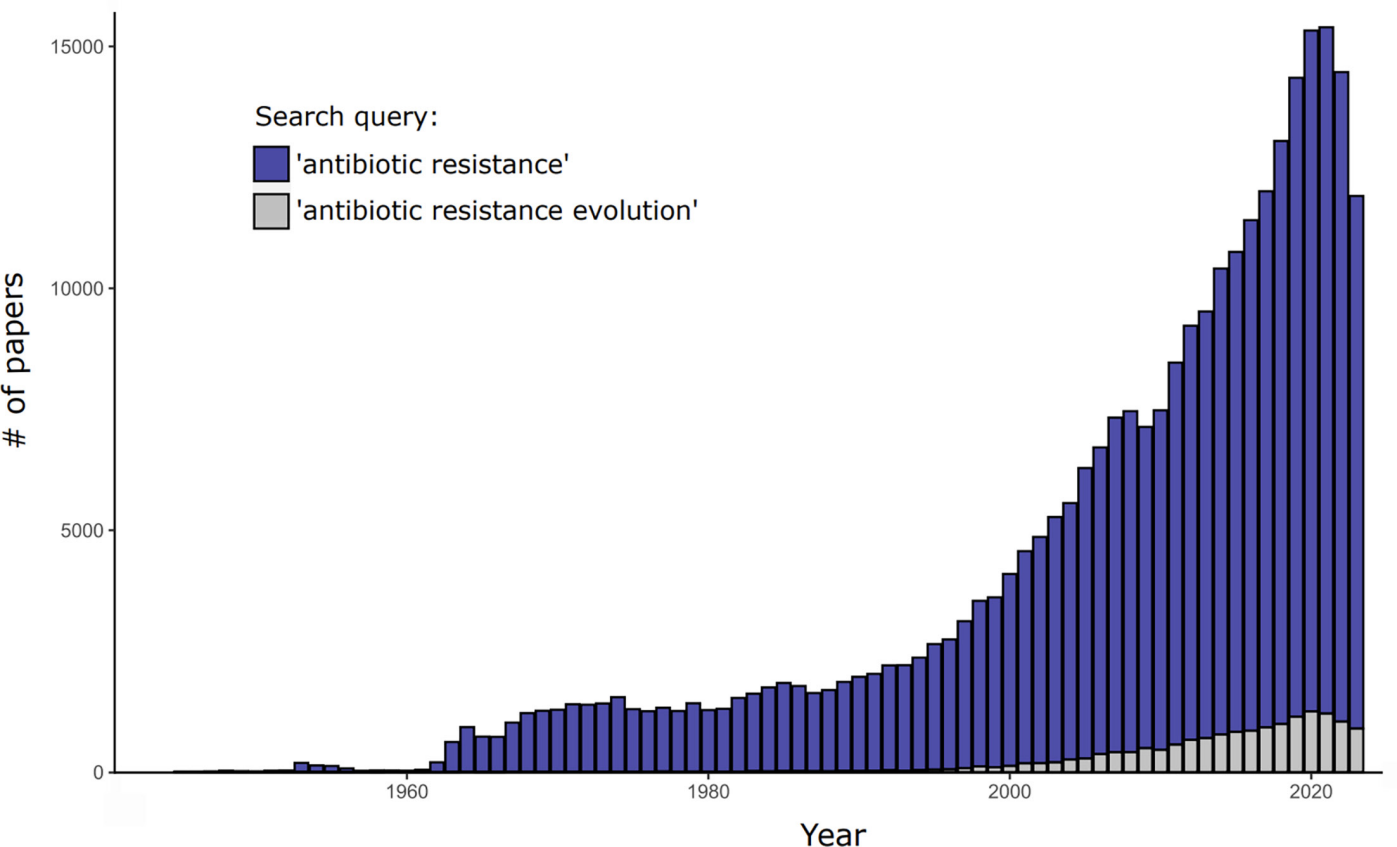
Publication trends reflecting evolutionary thinking about resistance. Number of PubMed papers associated with search terms ‘antibiotic resistance’ and ‘antibiotic resistance evolution’ each year, from 1945 to 2023. Blue bars include the grey in their counts. Note that these search terms are restrictive and hence could have excluded studies addressing resistance evolution in a less explicit manner.

The scale of resistance evolution should have already become apparent following the commercialization of penicillin by Chain and Florey in 1940 [10]. Global penicillin adoption and the attributed mortality reduction, amplified in the wake of World War II, cemented antibiotics as key contributors to improved public health, adding 20 years to life [10]. Consequently, as excessive usage became commonplace, resistance became widespread within years [11,12] – a vicious pattern that has repeated itself for almost all beta-lactams (BLs) (Fig. 1). And yet, only in recent years has the impact of AMR evolution begun to be evaluated in earnest. Whilst antibiotic resistance receives a dedicated research focus, it is typically studied outside its explicit evolutionary context (Fig. 2).

Since the discovery and commercialization of penicillin, novel antibiotics with differing mechanisms of action have been developed from both natural and synthetic precursors [13,14]. Mass use in humans and agriculture has imposed selection for resistance across bacterial species [15]. Factors shaping resistance evolution have been studied experimentally, bioinformatically and theoretically [16,18], in clinical [12,19], agricultural [20,21], environmental [22,23] and laboratory settings [24]. More recently, novel methods of data analysis, such as machine learning, have enabled researchers to link clinical behaviour to resistance evolution [25]. Consequently, key mechanisms that confer resistance are well-known for many antibiotic groups [26,27], as is their relationship to fundamental evolutionary properties such as mutation supply and strength of selection [28,29].

However, studies into resistance evolution often focus on immediate causes: for example, the effect of antibiotic concentration [12,30], the number of antibiotics used [31,32] or the evolutionary pathways to resistance under controlled laboratory or clinical conditions [16,33]. Comparatively, the association between resistance evolution and policy decisions from governments, industry and regulatory bodies is poorly understood, with the AMR benchmark score representing a contemporary effort in defining this relationship and its consequences [34].

Our aim is to identify major knowledge gaps regarding the consequences of stakeholder decision-making on resistance evolution by applying a historical and ontological approach to several case studies. In doing so, we provide insights into how evolutionary thinking can inform and be incorporated into antibiotic development, manufacture and administration policy. We will focus on BL antibiotics in humans, given their clear development timeline and continuous molecular refinement to meet emerging clinical and microbiological needs. At 65% of the total antibiotic market, they form the largest antibiotic group [35]. The widespread evolution of resistance to BLs (Box 1) poses a significant threat to mortality, quality of life [36] and global society [3].

Box 1: **BL resistance and beta-lactamases**

The Ambler system classifies beta-lactamases that confer resistance by enzymatic cleavage, the most common mechanism of resistance (Fig. 2). Other mechanisms such as modification of penicillin-binding proteins and the regulation of BL entry and efflux have also been observed [163].

A key differentiator relates to whether the active site contains a serine, the basis of the extended-spectrum beta-lactamases (ESBLs – classes A, C and D) or zinc ion at the centre, the metallo-beta-lactamases (MBLs – class B) [164]. Carbapenemases, which inactivate carbapenem BLs are found within classes A, B and D and include *Klebsiella pneumoniae* carbapenem(KPC)-resistant, MBL and oxacillinase (OXA)-type enzymes [165]. Carbapenemases are a major concern given their ability to render the majority of BLs ineffective [166]. Broad-spectrum beta-lactamases, including ESBLs and MBLs, are coded by the *bla* gene family. The *Bla* gene family forms the largest class of antibiotic resistance genes [167] and is responsible for the majority of antibiotic resistance occurrences [168].

Classification of beta-lactamases. Major enzymes from each of the four Ambler classes are depicted, with colours indicating the substrates hydrolysed by each enzyme. Adapted from [169].

## Case study 1: Research sharing accelerates drug development – the NRDC

In 1949, allied with the Development of Intentions Act, the UK Government founded the National Research Development Corporation (NRDC). Its mission was to incentivize publicly funded research and was ostensibly a countermeasure against the loss of penicillin rights to the USA [37]. Possibly inspired by Chain and Florey’s decision not to patent the process of large-scale penicillin production [38], the NRDC influenced key decisions that had considerable impact on the development, production and use of cephalosporins [39] (Box 2). Specifically, companies that initially developed and produced novel cephalosporins were required to share research findings with other NRDC licensees. These antibiotics were associated with significant refinements to efficacy, tolerance and administration, including oral use [39,40].

Box 2: **Cephalosporins**

In 1948, the same decade as penicillin commercialization, the first known cephalosporin was isolated in Sardinian sewage water by Guiseppe Brotzu. However, it was only a few years later that Edward Abraham and colleagues identified cephalosporin C (CPC) from this culture [170]. The BL ring in cephalosporins is bonded to a six-membered dihydrothiazine ring, in comparison with the penicillin BL ring, which is bonded to a five-membered thiazolidine ring [171]. The elucidation of the CPC structure in 1953 arose at a time when penicillin-resistant *Staphylococcus* emerged in hospital settings [172], creating a need for more effective treatments. As such, CPC became an important drug in its own right, as well as a precursor for the development of other cephalosporins including cephalosporins P and N [173] that were active against a similar spectrum of bacteria as penicillin.

First-generation cephalosporins entered clinical use in 1964 [174] and offered advantages over penicillins, such as increased activity against penicillinase-producing bacteria [175,176] and a more favourable allergy profile, compared with penicillins [177]. Due to these advantages, cephalosporins became frequently used for treating hospital infections in the 1970s [175,178]. For example, penicillin-resistant strains of *Staphylococcus aureus* were found to be susceptible to cephalothin [179]. Similarly, third-generation cefamandole possessed greater Gram-negative activity and was introduced to overcome the emerging issues caused by *Enterobacter* infections [180]. There are now five generations of cephalosporins, which have realized various needs including improved oral availability [40] and efficacy against *Pseudomonas* [181,182].

The advantages of cephalosporins led to them being dubbed ‘wonder drugs’ [183]. Consequently, they were regularly used as first-line antibiotics, leading to increased demand [184]. It has been estimated that the first three cephalosporin generations delivered a staggering 50% reduction in post-operative infections in hospitals between 1960 and 1990 [39]. Cephalosporins, along with penicillins and carbapenems, accounted for 50–70% of overall antibiotic use across several European Union countries in the 1990s, illustrating the long-term reliance on BLs to treat and prevent infections [175,185].

An almost immediate consequence of the incentivized sharing of research findings was the incitement of exploratory research and novel modes of funding. The extensive shared research between companies thrust development throughout the 1950s [39,41]. In fact, the UK research sector, in part as a consequence of NRDC, stimulated the development of first-generation cephalosporins on a global scale, especially across Europe, the USA and Japan [42].

**Fig. 3.**
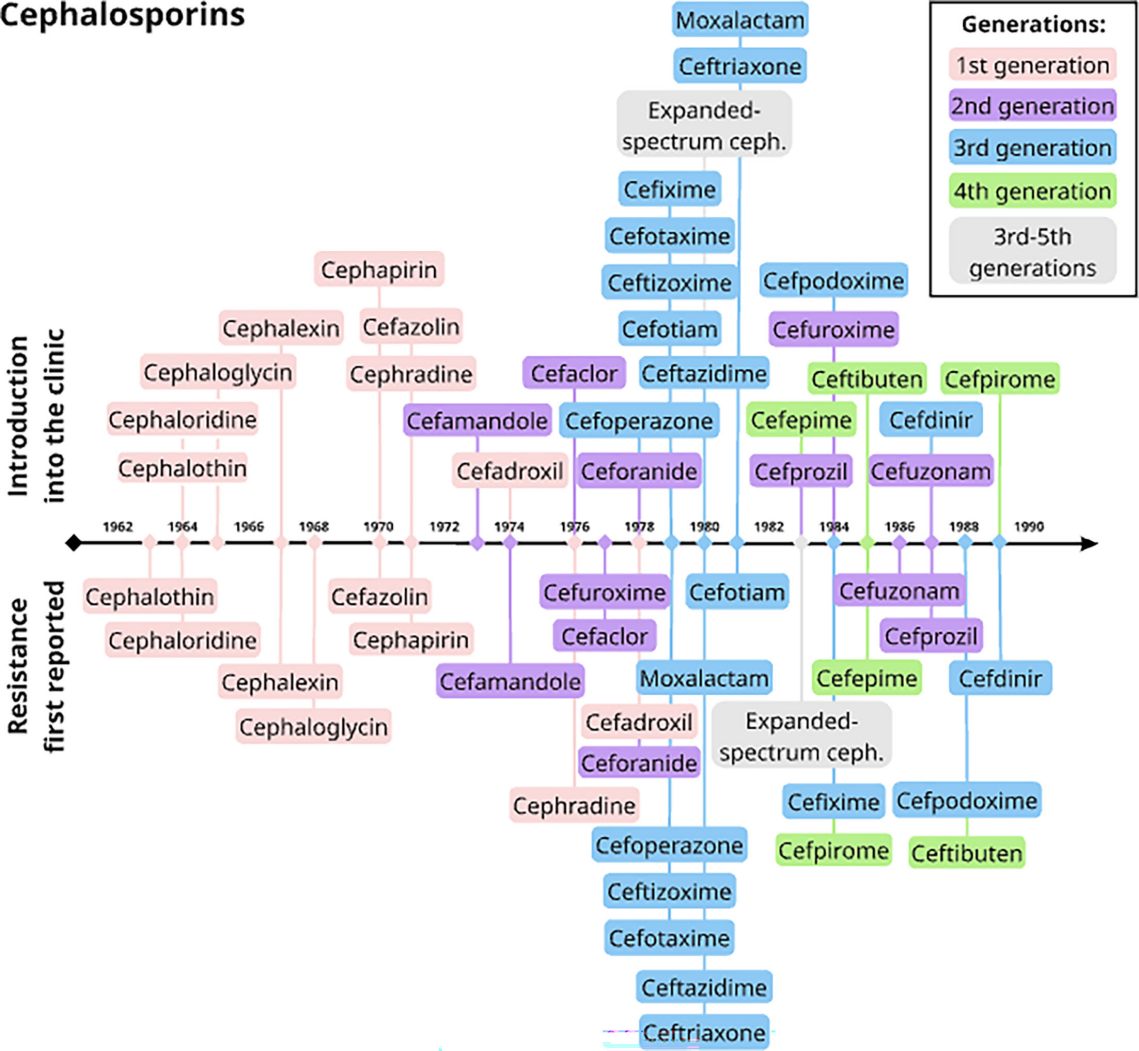
Timeline of cephalosporin introduction into clinical use and the first reports of resistance. The same methodology was used as in Fig. 1.

However, the evolutionary consequences of the rapid influx of novel therapeutics into clinics and farms throughout the mid-1950s and early 1960s were not clearly prioritized by the NRDC and its stakeholders. Novel drugs were approved for clinical use soon after development [43], and their use was poorly stewarded [5] without consideration of the potential for cross-resistance and horizontal gene transfer [44]. Together, these factors likely promoted resistance evolution, yielding resistance to individual BLs as well as other drugs through cross-resistance. The first known cephalosporin-resistant bacteria were isolated as early as 1968, shortly after their clinical adoption (Fig. 3) [45,48]. This trend continued into the latter half of the century, with resistance sometimes arising even prior to clinical use [48].

*Enforced sharing of research findings between shareholders in the private and public sectors stimulatedthedevelopment of novel antibiotics. However, a lack of consideration and contingency for stewardship and AMR evolution (including surveillance, data sharing and translation of lab findings to the bedside) likely compromised the NRDC’s ability to ensure sustainable, long-term effectiveness of antibiotics*.

## Case study 2: Medical and financial drivers of prescription volume – beta-lactamase inhibitors

In the early 1980s, co-amoxiclav (Box 3) was approved in the UK and the USA [49,50] and soon entered the WHO list of essential medicines [51,52]. It was licensed for community-acquired pneumonia (CAP) and urinary tract infections (UTIs) and continues to be one of the most consumed antibiotics globally [53,54]. By 2001, some regions treated 90% of CAP cases with this combination of amoxicillin (BL) and clavulanic acid [beta-lactamase inhibitor (BLI)] [55,56]. It was also effective against Gram-negative extended-spectrum beta-lactamase (ESBL) infections [57] – a significant feature given the progressive rise of enzymatic resistance over this time [58]. Co-amoxiclav was perceived favourably and adopted at scale largely due to its tolerability and broad-spectrum activity [59]. In addition, it provided the ability to directly switch from intravenous to oral treatment, further boosting its appeal [60].

Box 3: **Drug combinations using BLs and BLIs**

Resistance to penicillin was already widespread by the early 1950s, with 50% of hospital isolates caused by penicillin-resistant *Staphylococcus aureus* [186]. Resistance occurred through the inactivation of the BL ring [187], sparking the search for an inhibitor of beta-lactamase enzymes, initially with little success. Once identified, it became apparent that co-administration with a BL antibiotic prevented cleavage by Gram-negative bacteria and restored drug activity [188].

The first BL/BLI combination introduced in 1981 was co-amoxiclav – a combination of amoxicillin and clavulanic acid [189]. Clavulanic acid, which contains a BL ring (i.e. a BLI with a BL ring), was isolated from *Streptomyces clavuligerus* in 1970 [190,191] and lacked significant intrinsic antimicrobial activity. However, when combined with amoxicillin, it lowered the MIC of amoxicillin against pathogenic bacteria such as *Klebsiella pneumoniae* and *Staphylococcus aureus* [192,193]. Combinations such as ampicillin/sulbactam and piperacillin/tazobactam (TZP) soon followed [194,195], all showing a similar ability to control the spread of ESBL-producing organisms. More recently, non-BL BLIs, which do not contain a BL ring structure, were developed to overcome further resistance issues.

During this period, the semantics of ‘strong(er)’ antibiotic (in contrast to the modern-day thinking that moved towards ‘appropriateness’) were embraced, so that the perceived benefits of co-amoxiclav led to patients being treated for the same infection with the same drug multiple times [61] – driving resistance evolution [60,62]. Consequently, we now witness over 40% resistance in *E. coli* isolates in the UK [63].

Following the commercial success of co-amoxiclav, new BL/BLI combinations were developed. Piperacillin, an extended-spectrum penicillin, was combined with the BLI piperacillin/tazobactam (TZP) and approved for use in 1993 [64]. It possesses activity against beta-lactamases, penicillin-resistant bacteria [65] and *Pseudomonas aeruginosa* with licensed indications for hospital-acquired pneumonia (HAP), intra-abdominal infection (IAI) and skin and soft tissue infection [66]. Consequently, TZP was ubiquitously adopted, becoming the ‘workhorse antibiotic’ responsible for 3.6% of global antibiotic use in 2009 [67]. Similar to co-amoxiclav, TZP is subject to repeat and inappropriate use [68], which has continued despite surveillance studies frequently identifying TZP resistance in *Enterobacterales* [63] and *Pseudomonas* [69].

*Marketing forces pushed only a small number of antibiotics, narrowing the diversity of potential prescription patterns and compromising stewardship. These antibiotics still provide essential roles across the globe,and with stewarded use,they realize critical areas of need. However, their mass adoption has contributed significantly to the current resistance problem,and their use should be rationalized*.

## Case study 3: The cephalosporin era – short-term market forces and their consequences

The clinical success of cephalosporins [70] (Box 2) resulted in sharply increasing sales under bold marketing [39,71]. Ten years after the first cephalosporins were introduced into clinics, their first generation had annual sales of $640 million [39]. The increasing trend continued, with annual sales reaching $1 billion by 1984 [39,71]. Prominent marketing led to the development and clinical use of the next generations of this drug class [72,75] and to increased revenue and market share [64,76].

ESBLs form the class A group of beta-lactamases (Box 1) [65] with hydrolytic activity against cephalosporins and penicillins [77], rendering them ineffective against Gram-negative bacterial infection. Significant concern regarding resistance arose with the emergence of ESBLs in 1983 [78,79], particularly as they became a common cause of hospital-acquired infections and associated mortality in critically ill patients [80,82]. Today, ESBL outbreaks have become commonplace [83,84], with over 200 enzyme types having been identified [85]. This rapid emergence and spread of resistance was likely driven by mass use and the simultaneous introduction of many drugs with similar mode of action within a short interval [39,86, 87].

A silver lining is that, as ESBL resistance evolution gathered pace, it prompted what is considered to be perhaps the first iteration of antimicrobial stewardship (AMS): to combat the spread of ESBLs, methods such as antibiotic streamlining [88] (and restricted cephalosporin use [89] emerged between 1988 and 1990.

*Cephalosporins continue to provide a backbone of modern infection management. However, their application was compromised by poorly stewarded use: short-term market forces impacted long-term effectiveness and value by not accounting for factors that stimulate resistance evolution*.

## Case study 4: Dead on arrival? Resistance evolution before an antibiotic is on the market

For several BL groups, resistance predates their clinical adoption (Figs2, 3  4) [90]. For example, metallo-beta-lactamases (MBLs), capable of hydrolysing diverse BLs including carbapenems (Box 4), were first detected a decade prior to the discovery of the first carbapenem in 1966 [91,92]. This was the first example to illustrate that the derivation of drugs from naturally occurring precursors might be accompanied with already evolved resistance. This MBL, BcII, was identified in *Bacillus cereus* [91,92] and was considered an enigma as it was discovered in soil bacteria [93,94].

Box 4: **Carbapenems**

The discovery of the first carbapenem, thienamycin, in 1976 [196] gave rise to a series of modified derivatives such as imipenem, the very first carbapenem, which entered clinical use in 1985 [197]. Increased interest surrounded it and its subsequent synthetic derivatives, meropenem and ertapenem [198], due to their stability against beta-lactamases and particularly ESBLs [199]. Carbapenems have a broad spectrum of activity against Gram-positive and Gram-negative bacteria.

Imipenem, administered in combination with cilastatin, and meropenem have activity against *P. aeruginosa*, a critical and hard-to-target organism. Both are used for the treatment of severe infection, such as IAI, HAP and UTIs [200]. However, carbapenems do not have activity against methicillin-resistant *Staphylococcus aureus* (MRSA) or *Enterococcus* spp. [201]. The broad spectrum of Gram-negative activity sparked their widespread use for treating severe infections, despite their intended placement as a ‘last-resort’ treatment [202]. The widespread use led to the evolution of resistance, which has been observed to (almost) all existing carbapenems [201].

**Fig. 4.**
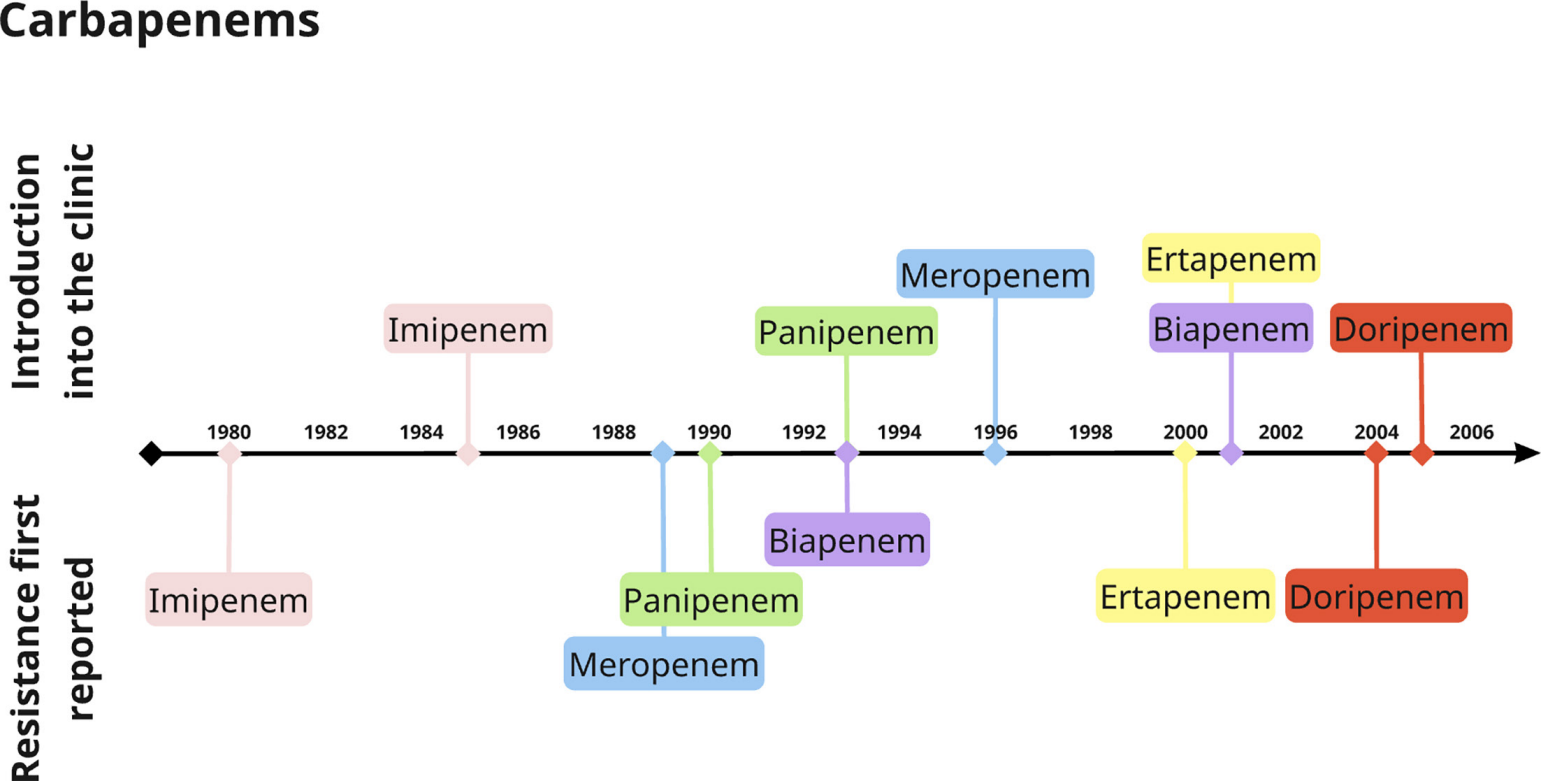
Timeline of carbapenem introduction into clinical use and the first reports of resistance. The same methodology was used as in Fig. 1. Colours indicate the antibiotic.

Interestingly, BcII was the only MBL to be discovered for the next two decades, until the cassette chromosome recombinase A protein (CcrA) was discovered in *Bacteroides fragilis* in 1980 [95]. This enzyme, encoded by the *bla*_CcrA_ gene, hydrolyses almost all known BLs and, crucially, is not inactivated by BLIs such as clavulanic acid [96,97]. Several other enzymes, such as *Klebsiella pneumoniae* carbapenem (KPC) and New Delhi metallo-beta-lactamase (NDM), with a similarly broad spectrum of activity were identified soon after, presenting a significant threat to the efficacy of most BLs [98].

Despite the academic finding in 1966 and the emergence of MBLs in 1980, carbapenems were still used at mass scale throughout the 1990s. This heavy use created a strong selection for resistance, which could spread even faster when mechanisms of resistance are already present in bacterial populations [99]. Consequently, a series of class A, B and D beta-lactamase gene variants were identified (Box 1). For example, imipenem-resistant *Bacteroides fragilis* was identified in 1986 [100], with imipenem entering clinical use in the same year.

The spread of resistance was further accelerated as resistance genes incorporated into plasmids – mobile genetic elements that can be horizontally transferred between strains, including between unrelated bacterial species. Plasmid transfer of existing resistance genes was a major contributor to the rise and spread of AMR in South America throughout the 1990s and 2000s, with a substantial portion of *P. aeruginosa* isolates containing MBLs [101]. Rapid plasmid proliferation can also accelerate their subsequent evolution [102], a potential explanation for the emergence of the NDM – an MBL that could hydrolyse all known BLs at that time [103].

Carbapenems have been favoured for their pharmacokinetic profile, tolerance, wide spectrum of activity and clinical efficacy [104]. However, decision-makers during the peak use of carbapenems did not adequately account for the existence of resistance in natural populations, nor the potential for horizontal gene transfer to exacerbate the spread and subsequent evolution of resistance genes. Interestingly, despite the rapid resistance evolution observed in the last decades of the twentieth century, carbapenems remain viable in many parts of the world – pointing to the importance of stewardship in preserving the efficacy of antibiotics. Today, we know that genes for synthesizing beta-lactamases are ancient, likely predating the split between Gram-positive and -negative bacteria [105,106]. Resistance to BLs is just as old [107,108] and can be found in permafrost soils that have been entirely free from human contact [109]. And yet, drug development pipelines do not typically account for the potential presence of resistance in natural bacterial populations. This is not surprising, given that we lack a framework to guide decisions on whether to proceed with developing a lead compound into a clinically relevant antibiotic, when resistance to it is found in the environment.

*Resistance to pipeline drugs can already exist in bacterial populations, especially if they are derivatives of natural precursors. The spread of resistance to a new drug is accelerated when it is already present in the population, which has in the past compromised the use of carbapenems as a last line of defence. A clear appraisal of the resistance potential for pipeline drugs and deriving value beyond volume use would preserve their long-term value*.

## Case study 5: Limited options in the pipeline – consequences of concentrated manufacturing for the global supply of TZP

Whilst the awareness of resistance to most widely used drugs was causing increased clinical concern, fewer antimicrobials (including BLs) were being developed or entering clinical use [43,110] (Figs3  4). Most BLs entering clinical use since the 1990s have been combinations of existing drugs, such as piperacillin/tazobactam [111]. In the past, the development of novel drugs was outpacing the resistance evolution that could make them obsolete [39,89], but this was no longer the case. The reduced number of viable drugs has resulted in an increasingly dire situation, such as the availability of just a few BL/BLI combinations with activity against carbapenem-resistant *Enterobacterales* and multi-drug-resistant *Pseudomonas* [112].

Simultaneously, production and supply became more concentrated, typified by TZP, which was manufactured in only a few sites globally [113]. In 2016, one of these factories burnt down, leading to a major global shortage [114] of this essential workhorse antibiotic [115]. Clinicians were forced to use alternative treatments such as carbapenems, further exacerbating the evolution of carbapenem resistance [116]. This shortage was also linked to the rise of severe *Clostridium difficile* infections due to the use of other treatment options [117]. More recently, shortages of antibiotics across European countries [118] have risen sharply due to supply chain disruption and increased demand as a consequence of the COVID-19 pandemic [119].

*Centralization of production pipelines can increase the risk of antibiotic shortages, resulting in suboptimal treatment regimens. In this way, antibiotic production, if concentrated and not diversified, creates an ideal milieu for accelerated resistance evolution* [114].

## Case study 6: Targeted drug development for areas of need – a modern paradigm

BLIs that contain a BL ring, such as clavulanic acid, tazobactam and sulbactam, were developed to tackle the rise of ESBL-producing bacteria (Box 3). However, BLIs with a BL ring exhibit a narrow spectrum of activity, typically inhibiting only class A beta-lactamases (excluding KPC). Resistance to BL/beta-lactam-ring BLIs evolved throughout the 1990s [120], potentially enhanced by their narrow spectrum. To counter this and recover their clinical utility, the pursuit of non-BL BLIs (BLIs that do not possess the BL ring) commenced. In further contrast to previous drug development approaches, this time, the effort was pathogen directed and rooted in the understanding of antibiotic resistance mechanisms.

The first non-BL BLIs, such as vaborbactam and relebactam, were introduced in the 2010s [121,124]. They could inhibit class A (including KPC), class C and some class D beta-lactamases. In other words, this new class of drugs compensated for the reduced utility of BLIs with a BL ring, as it was specifically designed to do so. The development pipeline of non-BL BLIs also points to the benefits of using drugs with a diverse spectrum of activity, which can slow down resistance evolution by enabling diversification of drug use [125].

*Incorporating evolutionary thinking into drug design and candidate selection can enhance the short- and long-term effectiveness of antibiotics by increasing robustness against resistance*.

## The early stewardship era (1990–2010s)

Whilst the end of the century saw a reduction in antibiotic prescription rates, especially for second- and third-generation cephalosporins, extensive use of TZP, co-amoxiclav and carbapenems remained high [126]. It comes as no surprise that resistance continued to evolve and spread, necessitating coordinated, global actions.

One of the earliest such actions was the initiation of national and global long-term antibiotic resistance surveillance programmes, such as the SENTRY programme in 1997 [127]. SENTRY specifically arose as a reaction to the dissemination of ESBLs in clinical settings. In the late 1990s, the National Nosocomial Infections Surveillance (NNIS) system in the USA created subsets of surveillance programmes in intensive care units and surgical settings to monitor the spread of AMR [128].

Greater credence was placed on AMS, as it became a widespread term. Several policies emanated from AMR concerns (Table 1), and by 2006, all NHS trusts in the UK were mandated by statutory laws to adopt antimicrobial prescribing policies [129]. The United Nations initiated a global response in 1995, emphasizing multiple strategies to reduce the impact of AMR, many of which required implementation at a local level [130] (Table 2).

**Table 1.** Official UK regulations and guidance to improve antibiotic prescribing and stewardship, adapted from Ashiru-Oredope *et al.* [126]

Year	Author	Publication title	Focus	Type
1998	Standing Medical Advisory Committee (SMAC) sub-group on AMR	The Path of Least Resistance	Report on AMR	Report/recommendations
			National strategy for minimizing the development of AMR	
1999	Department of Health	Resistance to antibiotic and other antimicrobial agents: action for the NHS following governments response to the House of Lords Science and Technology Select Committee report ‘Resistance to antibiotic and other antimicrobial agents’	Set out action plan for the NHS, aimed at reducing the emergence and spread of AMR and its impact on the treatment of infection; includes strategies to monitor and optimize prescribing	Health Service Circular
2000	Department of Health	UK AMR Strategy and Action Plan	UK action plan to reduce resistance	Guidance
2003	Department of Health	Winning ways: Working together to reduce Healthcare Associated Infection in England	Set out for the first time a clear direction for the local NHS to reduce Healthcare Associated Infections (HCAIs); includes seven action areas, such as prudent use of antibiotics	Guidance
	Department of Health	Hospital Pharmacy Initiative for Promoting Prudent Use of Antibiotics in Hospitals	Letter highlighting new funding for promoting prudent antibiotic prescribing through enhanced clinical pharmacy activity	Chief Medical Officer Professional Letter
2006	Department of Health	The Health Act 2006	Code of practice for prevention and control of healthcare-associated infections; requires all NHS trust to have antimicrobial prescribing policies	Code of practice
2007	Department of Health	Saving Lives: reducing infection, delivering clean and safe care	Provides the tools and resources for trust; implementation of code of practice for prevention and control of healthcare-associated infections	Guidance/toolkit
	Specialist Advisory Committee on AMR (SACAR)	Antimicrobial Framework	A framework to support the safe and appropriate use of antimicrobials	Best practice, care guideline
2008	Department of Health	Health and Social Care Act 2008	Sets out what registered providers of health and social care services should do to ensure compliance with the Care Quality Commission registration requirement for cleanliness and infection control; includes antimicrobial prescribing and stewardship as guidance for compliance	Code of practice
2009	Department of Health and HPA	*Clostridium difficile* infection: how to deal with the problem	10 key recommendations, including AMR stewardship, for healthcare providers and commissioners to best tackle *C. difficile* infections	Guidance
	National Pharmacy Reference Group	Antimicrobial stewardship: an evidence-based antimicrobial self-assessment toolkit (ASAT) for acute hospitals	A web-enabled, version-controlled instrument for the assessment of AMS in acute hospitals	Self-assessment toolkit
2010	HPA	HPA Antibiotic Guidance for Primary Care	Updated antibiotic guidance for primary care clinicians, modifiable locally by PCTs and distributed to practices	Guidance

**Table 2.** WHO strategies for containing the emergence and transmission of antibiotic-resistant bacteria, adapted from Smith and Coast [130]

	Level of intervention			
	Local	National	Regional	Global
**General strategies for containing emergence and transmission of AMR**				
Surveillance	Required at all levels in order to obtain an accurate picture of emergence resistances and the rate of transmission of new resistances and to identify the impact of interventions designed to contain AMR in particular contexts			
Financial incentives or disincentives	Could be used at all levels in conjunction with many other policies as a mechanism for improving update of or compliance with any intervention; would include such mechanisms as financial benefits, environmental taxes and use of permit systems			
**Strategies for containing the emergence of resistance**				
Education of professionals	On specific problematic micro-organisms in local areas	On issues most relevant to general national conditions	On general principles through regional organization and the Internet	On general principles through global organization and the Internet
Education of patients on inappropriate use and compliance with instructions	Local campaigns and education by health professionals	By providing national information campaigns	On general principles through regional organizations and the Internet	On general principles through global organization and the Internet
Rapid diagnosis of bacterial infections	By improving local facilities	By providing infrastructure for improved local facilities	Through the provision of aid to countries whose infrastructure is lacking	Through the provision of aid to countries whose infrastructure is lacking
Control of sensitivity data related to prescribers	From local facilities	By providing infrastructure for improved local facilities		
Antimicrobial policies	Development of local facilities taking account of specific local conditions	Development of national medical associations		
Antimicrobial cycling	At local level, taking account of prevailing local conditions			
Regulation of antimicrobial use in agriculture		Developed by national policymakers	Through regional agreements	Through international agreements
Choosing the optimal agent, dose and dosage frequency	At patient level			
Removal of potential septic foci/prostheses	At patient level			
Use of drug combinations	At patient level			
Using antiseptics as an alternative	At patient level	Guidelines suggesting the use of alternative agents		
Using cranberry juice as an alternative to antibiotics for UTIs	At patient level	Guidelines suggesting the use of alternative agents		

A combination of these strategies alongside the prudent use of BL/BLI combinations [131,132] solidified the importance of mitigating resistance, particularly given the lack of novel antibiotic options on the horizon. Interest in resistance evolution increased (Fig. 1), but academic findings have not translated clearly into stewardship initiatives.

Despite their immense clinical value and enablement of modern expensive invasive procedures, poor return on investment [133] and a broken economic model [134] severely impacted investment into the development of novel antimicrobials, which declined significantly since the 1990s [135,136]. Investment is undermined by the inevitability of resistance evolution, which reduces the long-term efficacy of any potential new products [136]. Such problems are rooted in outdated economic and industrial models, and in particular, older development pipelines did not adequately account for factors that could mitigate the spread of resistance [137].

## Contemporary global initiatives (2010s to present)

Stewardship and policy reform have continued with vigour, and we now see action to combat AMR across the world. A landmark achievement by the WHO was the introduction of AMR national action plans, which have now been implemented in 178 countries [138]. Of particular importance was the introduction of the WHO priority pathogen list in 2017, which includes a considerable number carrying BL resistance. Detection and surveillance are vital and provide early signals of emerging resistance and pandemics [139]. The Global Antimicrobial Resistance and Use Surveillance System (GLASS) was launched by the WHO in 2015 to standardize data collection [140]. Efforts to combine stewardship and surveillance are gaining increased attention, as they have a major role to play in responsible and appropriate antibiotic use.

Dame Sally Davies spearheaded the first United Nations General Assembly (UNGA) AMR meeting in 2016, leading to 193 countries signing a declaration pledge to combat AMR [141]. This appears to have been the necessary touchpaper for action and the creation of initiatives and organizations committed to tackling the AMR crisis. GARDP, established in 2016, looks to bolster the pipeline and includes public, private and non-profit partners to create new treatments and reduce access inequalities [142]. Similarly, CARB-X emerged in 2016 in partnership with the UK Department of Health and Social Care and is a non-profit that aims to expand the antibiotic pipeline [143]. The AMR Industry Alliance (AMRIA) [144] represents the industry in its AMR efforts and seeks a collective effort from its members. The British Society for Antimicrobial Chemotherapy (BSAC) has been a longstanding player in the game and acts with global intent to advocate for better infection management. Their GAMSAS programme is underway to provide accreditation of standard AMS practice [145], permitting a means for commissioning-based approaches, incentives and reimbursement for stewardship as a distinct expert clinical activity. Together, these form prime examples of the collaborations necessary across governmental bodies and R and D organizations to target the resistant bacteria that pose the greatest threat to global health [146].

The AMR benchmark report exploits the putative, multifaceted approaches necessary for tackling AMR [34] by evaluating manufacturers and identifying key areas of need and achievement. The report highlights the importance of stewardship and pipeline developments that respond to issues of unmet need [146]. This is indicative of a worthy shift from producing treatments for syndromes of infectious diseases to pathogen-directed therapy and appraisal. The report also identifies the dangers of global inequality in access, prescription and use of antibiotics, positing that infrastructure improvements are a key requirement to reducing the global burden of AMR [147].

If novel antibiotics, including BLs, are to remain a cornerstone of therapy, their pipelines must remain innovative, sustainable and accessible. Today, new economic models are being instigated to stimulate the dwindling market through push-and-pull incentives. An example is the UK de-linkage model, an archetype of value-based healthcare, which provides reimbursement for antimicrobials based on the value they provide to healthcare rather than the volume used [148]. This is an example of a pull mechanism that incentivizes both responsible use and the development of new antibiotics. A vital aspect of such programmes is the incorporation of surveillance of AMR and prescribing practices into economic models, as done by the Antimicrobial Registry (UKAR) in the UK [149] and similar programmes in Sweden [150], Canada [151], Japan [152] and Switzerland [153]. A similar pull incentive, the PASTEUR Act, is being considered for adoption in the USA [154]. Some European states are considering adopting a different type of model, which offers patent exclusivity for any drug in their portfolio if they market an antimicrobial [155].

## Concluding themes

In this review, we focused on important historical case studies that shaped the development, use and long-term efficacy of BL antibiotics. We demonstrated important advances in science and discovery and how legislative and industry stakeholders have worked together to influence the direction of travel. However, some of these early actions exacerbated the problem that we face today because stakeholders did not fully consider the long-term, evolutionary consequences of their decisions. Modern technology offers a means of empowering evolutionary theory and a path towards better industrial and clinical practice that mitigates resistance spread, and to a world where antibiotics maintain their efficacy and true value for as long as possible. Novel thinking has led to redefining working antimicrobials as a common pool resource, that is, as a key utility of societal infrastructure. This definition allows for improved valuation of the societal value of antimicrobials and is fitting for both high- and low-income countries.

A recurring theme across the case studies has been the inevitability of resistance emergence and a time lag to implementing stewardship and containment strategies. In the past, resistance was rarely considered a problem until severe infections became untreatable. The NRDC demonstrated the value of stakeholder cooperation in stimulating rapid research and development but was not able to embed a cohesive stewardship and surveillance approach. The cephalosporin discovery era staged a time of rapidly evolving AMR, but the continuous influx of novel drugs delayed the understanding and appreciation of the magnitude of the problem. Consequently, towards the end of the nineties, investment into R and D of new antimicrobials became less attractive. A lack of diversity in discovery and production is typified by the case of the fire impacting global TZP supply. We hope that identifying, and acting to ameliorate, the impact of stakeholder decision-making on the emergence and spread of resistance can bolster investor confidence in antibiotic development.

The examined case studies yield important lessons too. For example, the rise of ESBLs led to the need for novel drugs. But, unlike in the past, the development of new drugs this time around accounted for pharmacological and clinical needs, resulting in the success of non-BL BLIs. For the first time, discovery pipelines also accounted for how drug properties might impact resistance evolution. Diversity in the antibiotic armoury allows diversification of drug use and plays a critical role in slowing down the emergence and spread of resistance [156]. Similarly, the use of BL/BLI drug combinations, as opposed to relying on individual drugs, marked a key milestone.

The advent of global coordinated actions and the formation of multi-disciplinary stakeholder organizations and initiatives is a critical turning point. These efforts are primarily focused on linking various disparate stakeholders to evaluate the problem in a global context. Others examine how the socio-political landscape and inequalities contribute to the problem. And some attempt to invoke a sustainable incentive for antibiotic development and stewarded use.

However, microbes and their resistance mechanisms do not respect geographic boundaries, creating a need for coordinated and effective actions across national, regional and global scales (Table 2) that are not met by existing efforts. There are many stakeholders in the current landscape, and although most policy seeks similar aims, there is a need for cohesion and common goals. Steps towards better integration of stakeholders across the pharmaceutical industry, government and regulatory bodies are critical and must be adopted on a global scale. This is why it is vital to recognize that the success of an incentive depends on several critical factors: (i) that all G20 countries contribute investment [157], (ii) that this investment and security incentivize drug development and (iii) that antimicrobials are accessible across the world.

The vital role that antimicrobials play in the world must be communicated as best as possible to realize true civic value and responsible use. AMR now has a OneHealth policy agenda, which spans humans, animals, food security and the environment. We witness experts from diverse disciplines pull together in an effort to curb its impact, with 2024 staging the third UN General Assembly meeting on AMR [158]. We argue that steps towards better integration of evolutionary theory across the pharmaceutical industry, government and regulatory bodies are critical and must be adopted on a global scale.

Table 3 summarizes current practice, persisting gaps and opportunities by case study theme for incorporating resistance evolution theory and thinking. Whilst important for existing drugs under extensive use, resistance surveillance must extend to drugs in development. This is particularly important as most drugs in development are derivatives of natural precursors, potentially leading to pre-existing resistance in bacterial populations. Applying dynamic and close-to-real-time surveillance is likely to yield major advantages in tackling the emergence and spread of AMR. Surveillance for pre-clinical candidates should be accompanied with better understanding of the relationship between the presence of resistance before the introduction of the drug and its spread once the drug is introduced. However, standing variation for resistance is not the only determinant of spread, as the propensity for resistance to evolve *de novo* is key. Experimental evolution [16] offers methodologies to test how rapidly resistance to novel drugs, and those in development, might emerge. These methodologies can also be used to evaluate how prescription strategies might impact resistance evolution, informing the choice of key parameters such as dosage [159,160] or drug combinations [161,162]. New laboratory, genomic and bioinformatic techniques are continually developed to improve the study and surveillance of resistance evolution, but they are rarely implemented into drug development pipelines and legislation.

**Table 3.** Current practice, persisting gaps and opportunities for resistance evolution theory application by case study theme

Case study theme	Current policy and behaviours	Persisting gap	Placement of applied evolutionary theory
*Case study 1:research and insights sharing*	WHO priority pathogen listWHO global research agendaSurveillance programmes*Public–private partnerships, e.g. GARDP and CARBX	Pipelines for similar medicines require stewardshipPersistent focus on similar mechanisms of activityA balance of free market and targeted need using value-based economics to de-risk pipelinesLocalized priority pathogen lists	Mapping the therapeutic and resistance landscape of pipeline drugsEmbracing OneHealth learnings for zoonotic spread and resistance overspillIncorporating evolutionary theory into pipeline due diligenceModelling of future resistance for individual agents under realistic scenarios
*Case study 2:drivers of prescription volume*	Volume de-linked economic model in the UK (or other similar models)AMS initiativesWHO National Action PlansEducation of public and healthcare providers	Incentivizing stewardship by considering working antimicrobials akin to societal infrastructure and through value-based economicsStewardship of generics on a global levelGaps in surveillance of global drug use and supply chainsAdoption of pull incentives with AMS at coreNeed for diagnostic stewardship	Dynamic and predictive linking between volumes of use to resistanceAdaptive and dynamic antibiograms, from lab to bedsideModelling of resistance and diagnostic needs to be incorporated into prescription practices
*Case study 3:market crowding on limited range*	Diversification in drugs developedConsideration of other antimicrobial technologies, alone or together with antibiotics	Sales of novel medicines predominantly limited to high-income countries, leading to overuse of a limited range of antibiotics in low-income countriesLack of clarity and detail in global consumption volumes	Inform future horizon and development pipelines by geographical needInform launch strategy and combination strategiesInform adjunct technologies, e.g. bacteriophageModelling of volume impact on resistance
*Case study 4:resistance surveillance*	Many surveillance programmes – per drug, pathogen, resistance mechanism	Lack of harmonized and standardized approaches for surveillance (varies according to programme)Poor linkage of animal, environmental and human AMRSurveillance is rarely real timegastro-intenstinal (GI) carriage targeted screening in some systems	Anticipatory surveillance approachesHarness computational and AI power in the prediction of resistance spreadHarmonize and standardize reporting per pathogen/drug/mechanismAccounting for environmental resistance in patient spreadPredicting impact on microbiome carriage
*Case study 5:diversity of manufacture and distribution*	Companies diversifying manufacture and supply	Increasing knowledge of this issueInconsistent global distribution and productionDiversification of productionNeed for equitable and robustly mapped supply chainsInfrastructure definition of working antimicrobial alignment: adopting regional sustainable development in diverse regions and economies	Better understanding of how distribution impacts resistance spreadOptimizing supply chains to minimize selection for resistanceMapping the OneHealth impacts of new antimicrobials
*Case study 6:need-driven development*	Novel drugs and pipeline are more aligned to areas of need per pathogen and resistance mechanismPull incentive roll-outs	Poor mapping of entire pipelinesRisk of duplicate researchDifficult to predict future resistance issues for novel medicinesNeed to link pull incentives to early discovery and development	Intelligent drug design based on retrospective and future predicted resistanceExperimental evaluation of resistance under laboratory conditions when selecting candidates for further development

* Surveillance programmes: ATLAS, Ears-NET, SENTRY, VIVLI, GLASS, PROMED, MAPP, BDTool, SEDRIC, MICROBE and SMART.

In this review, we focused on critical historical case studies that shaped the development, use and long-term efficacy of BL antibiotics. We discussed important advances in discovery science and how legislative and industry stakeholders worked together (knowingly or not) to influence the direction of travel. However, some of these actions have exacerbated the problem that we face today because stakeholders did not adequately consider the long-term consequences of drug development pipelines and administration policies. Better integration between evolutionary microbiologists and key decision-makers offers a path towards a better industrial and clinical practice that minimizes resistance spread and, ultimately, a world where antibiotics maintain their effectiveness.

## Abbreviations

AMR: antimicrobial resistance
AMS: antimicrobial stewardship
BL: beta-lactam
BLI: beta-lactamase inhibitor
CAP: community-acquired pneumonia
ESBL: extended-spectrum beta-lactamase
HAP: hospital-acquired pneumonia
IAI: intra-abdominal infection
KPC: *Klebsiella pneumoniae* carbapenem
MBL: metallo-beta-lactamase
NDM: New Delhi metallo-beta-lactamase
NRDC: National Research Development Corporation
TZP: piperacillin/tazobactam
UTIs: urinary tract infections
